# Diverse Roles for a Conserved DNA-Methyltransferase in the Entomopathogenic Bacterium *Xenorhabdus*

**DOI:** 10.3390/ijms231911981

**Published:** 2022-10-09

**Authors:** Nadège Ginibre, Ludovic Legrand, Victoria Bientz, Jean-Claude Ogier, Anne Lanois, Sylvie Pages, Julien Brillard

**Affiliations:** 1 DGIMI, INRAE, Université de Montpellier, 34090 Montpellier, France; 2 LIPME, Université de Toulouse, INRAE, CNRS, 31320 Castanet-Tolosan, France

**Keywords:** Dam, methylome, MTase, *X. nematophila*, *X. kozodoii*

## Abstract

In bacteria, DNA-methyltransferase are responsible for DNA methylation of specific motifs in the genome. This methylation usually occurs at a very high rate. In the present study, we studied the MTases encoding genes found in the entomopathogenic bacteria *Xenorhabdus*. Only one persistent MTase was identified in the various species of this genus. This MTase, also broadly conserved in numerous Gram-negative bacteria, is called Dam: DNA-adenine MTase. Methylome analysis confirmed that the GATC motifs recognized by Dam were methylated at a rate of >99% in the studied strains. The observed enrichment of unmethylated motifs in putative promoter regions of the *X. nematophila* F1 strain suggests the possibility of epigenetic regulations. The overexpression of the Dam MTase responsible for additional motifs to be methylated was associated with impairment of two major phenotypes: motility, caused by a downregulation of flagellar genes, and hemolysis. However, our results suggest that *dam* overexpression did not modify the virulence properties of *X. nematophila*. This study increases the knowledge on the diverse roles played by MTases in bacteria.

## 1. Introduction

DNA methylation has been mainly studied in eukaryotes, where it is involved in cell differentiation or disease occurrence. The enzymes responsible for DNA methylation are called DNA-methyltransferases (MTases). They allow the addition of a methyl group directly to an adenine or a cytosine in the DNA molecule, using S-adenosyl-methionin as the universal methyl group donor [1]. In bacteria and in archaea, MTases usually recognize specific motifs on DNA, where three possible types of DNA methylation modifications have been described: N6-methyl-adenine (6mA also called m6A), C5-methyl-cytosine (5mC or m5C), and N4-methyl-cytosine (4mC or m4C) [2]. This process usually occurs shortly after the DNA replication in the growing cell, on the newly synthesized strand [3].

Bacterial MTases belong to restriction-modification systems [4,5] when they are associated with restriction endonucleases (REases) that protect the bacterial cell from exogenous DNA [6,7]. The DNA-methylation pattern settled by these MTases can thus allow the bacteria to discriminate between exogenous and endogenous DNA, then cause the degradation of foreign DNA by the cognate REase. In addition, “solitary” (also called “orphan”) MTases are also frequently found in the genomes of bacteria [5,8]. Some of these bacterial solitary MTases have broad conserved functions in genome maintenance (genome replication, mismatch repair, etc. [9]. One of the best characterized bacterial MTase is Dam (DNA-adenine methyltransferase), originally identified in *Escherichia coli* and widespread among Gammaproteobacteria [6,9]. This solitary MTase recognizes GATC motifs in the DNA, where it modifies the adenine in m6A. DNA methylation can also affect the interaction of DNA-binding proteins with their recognition sites, either by a direct effect (e.g., steric hindrance) or by changes in DNA topology [10], therefore causing epigenetic regulations [11,12]. Still scarce, evidence of bacterial epigenetic regulation are increasingly reported [2]. Such epigenetics regulations are responsible for prokaryotic phenotypic heterogeneity [12,13]. This phenomenon, where sister cells in a clonal population are differentiated in several populations displaying different phenotypes, is of critical importance for a successful infection by various bacterial pathogens [14,15].

Because of these diverse roles played by bacterial DNA-MTases, their deregulation (i.e., either an upregulation caused by the overexpression of their encoding genes, a downregulation, or even a lack of expression by the construction of a knockout mutant) can modify bacterial physiology, leading to broad and important impacts for the bacterial life cycles. Studies using such tools can therefore allow the identification of new phenotypes associated with these MTases. Most of them have been made in mammalian pathogens. For instance, Dam mutants of *Salmonella* Typhimurium are avirulent in mice [16,17], but Dam-overexpressing strains of *Salmonella* are also highly attenuated in mice [17]. Similarly, using Dam deregulated strains, the role of this MTase in virulence has been reported for other bacterial genera: *Actinobacillus* [18], *Aeromonas* [19], *Haemophilus* [20], *Klebsiella* [21], *Pasteurella* [22], *Vibrio* [23], or *Yersinia* [24]. Inactivation of the *dam* gene in some bacterial species was also shown or suspected to be a lethal mutation in *Yersinia pseudotuberculosis, Vibrio cholera, Aeromonas hydrophila*, and *Photorhabdus* [19,23,25,26], illustrating the fact that an alteration of the DNA methylation pattern can be of critical importance in bacterial physiology. Altogether, despite the description of broad roles played by bacterial MTases, in most of the studies cited above, no direct link between the targets of the MTase and the reported phenotypes was described [27,28].

Bacteria of the genus *Xenorhabdus* are pathogenic to a wide spectrum of insects [29]. These bacteria are also symbiotically associated with entomopathogenic nematodes that are used for biological control against insect pests of crops. After insect infestation, the nematodes release their bacterial symbionts into the insect host, leading to its rapid death by septicemia. We have previously showed that in *Photorhabdus*, a bacterium phylogenetically close to species of the genus *Xenorhabdus* and with similar lifestyle, the overexpression of the Dam MTase impaired various phenotypes, such as motility and virulence in insects [26]. Such overexpression also caused an increase in the rate of GATC methylated sites recognized by Dam [30]. Because these sites were frequently located in promoter regions, the hypothesis of epigenetic regulations linked to competitions between the Dam MTase and DNA-binding proteins at these particular GATC sites was raised [30,31]. In a study on more than 200 bacterial species, methylation marks were reported in one strain of *Xenorhabdus*: they were mapping in three motifs, including GATC methylated by Dam [5].

The purpose of the present study was to decipher the importance of DNA-MTases in *Xenorhabdus*. We first analyzed the distribution of putative MTases encoding genes among this genus, and showed that Dam was the main MTase conserved across the *Xenorhabdus* genus. By analyzing the methylome of two *Xenorhabdus* species, the most abundant methylated sites identified were mapping at GATC, recognized by Dam. The phenotypes associated with Dam overexpression were therefore analyzed and revealed diverse functions for this MTase in *Xenorhabdus*.

## 2. Results

### 2.1. MTases Repertoire in the Genus Xenorhabdus

**Figure 1:**
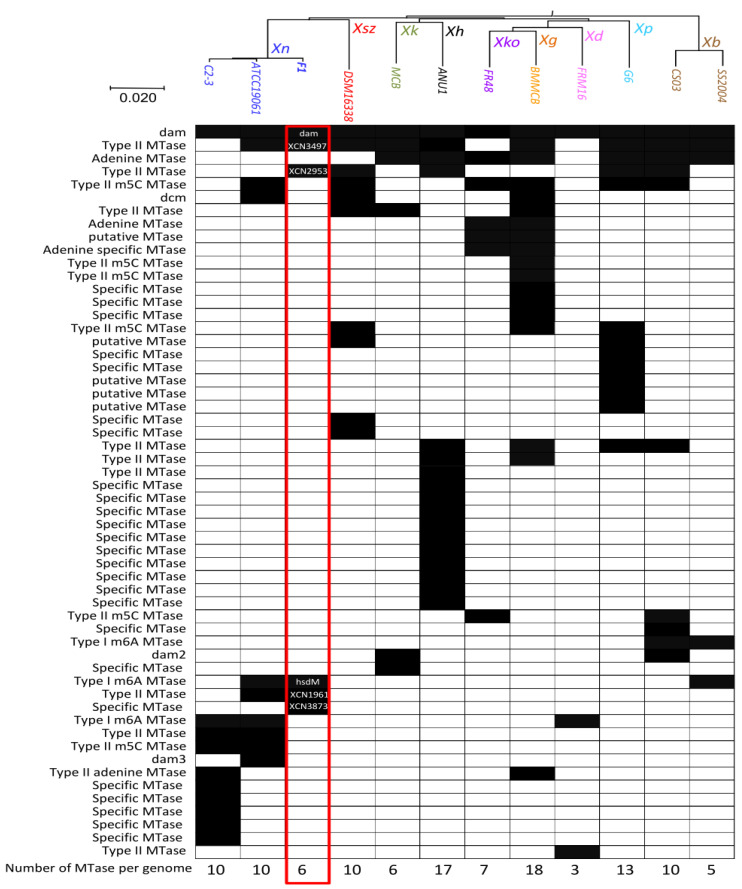
**Diversity of putative DNA-methyltransferase (MTases) in 12 *Xenorhabdus* genomes.** The distribution patterns of putative ortholog MTases among *Xenorhabdus* genomes is presented. Black square: presence of the MTase in the *Xenorhabdus* genome; white square: absence of the MTase. The phylogenetic tree (at the top of the figure) is based on complete *Xenorhabdus* genome sequences (see Materials and Methods section for details), *Xenorhabdus* species are color-coded, and the corresponding abbreviations are indicated (*Xb*, *X. bovienii*; *Xd*, *X. doucetiae*; *Xg*, *X. griffinae*; *Xh*, *X. hominickii*; *Xk*, *X. khoisanae*; *Xko*, *X. kozodoii*; *Xn*, *X. nematophila*; *Xp*, *X. poinarii*, and *Xsz*, *X. szentirmaii*). The number of MTases per genome is indicated at the bottom of the figure.

The description of the MTases repertoire among the *Xenorhabdus* genus was performed by extracting data from the REBASE database [7]. It includes 12 strains from 9 different *Xenorhabdus* species. The distribution of MTases genes identified by BlastP analysis among these *Xenorhabdus* species, ordered in a phylogenetic tree made by Average Nucleotide Identity (ANI) analysis (see the Materials and Methods section for details), is presented in Figure 1. The mean number of MTase encoding genes per genome was 9.8. Because some MTases have two distinct subunits, the number of MTase encoding genes can be higher than the estimated number of MTases. Taking this into account, the mean number of MTases per genome was 9.4. However, the distribution varies substantially between the strains studied here, ranging from 3 putative MTases in *X. doucetiae* to 18 in *X. griffinae*. Most of the DNA-MTase encoding genes were specific to one or a few strains of the *Xenorhabdus* genus (Figure 1). Our analysis revealed that only one of the MTase encoding genes was conserved among all the *Xenorhabdus* strains investigated (XNC3v3_0322 in *X. nematophila* F1 strain), and therefore could be qualified as a persistent MTase. This MTase, which is also broadly conserved in other Gram-negative bacteria, is annotated as Dam (DNA-adenine methylase) in the REBASE. In *Xenorhabdus*, this Dam MTase therefore most likely methylates adenines found in GATC motifs, as experimentally observed in other bacteria.

### 2.2. Methylome Analysis in Xenorhabdus

**Figure 2:**
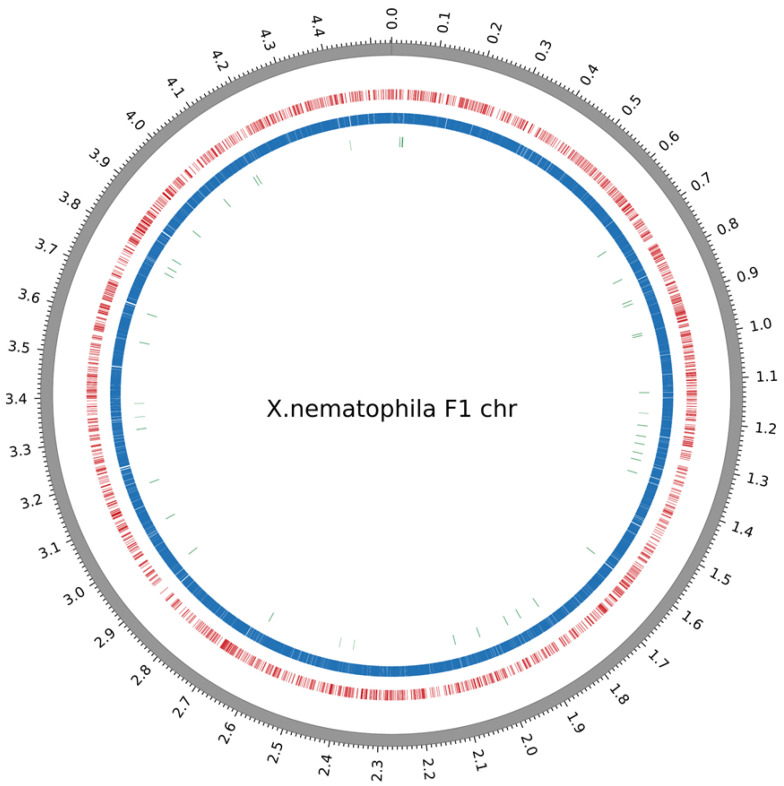
**Circos plots displaying the distribution of methylated bases over the *X. nematophila* F1 chromosome**. Outermost track displays the genomic positions in megabases. The colored tracks display the location of the modification marks detected, for each of the 4 identified motifs. From outer to inner: red, CAGNNNNNGTG/CACNNNNNCTG; blue, GATC; green, AANNNCCGGGNNNNNGA.

The methylome analysis of our model strain *X. nematophila* F1 was performed after SMRT (single-molecule real-time) sequencing by PacBio. An average sequencing coverage of 128X was reached, allowing the identification of a high number (n = 35,846) of statistically significant (QV score ≥ 30, see the Materials and Methods section for details) DNA modification marks. These methylated nucleotides were mapping in four motifs (Table 1). Three of them displayed m6A modifications and one displayed an m4C modification. The GATC motif, known to be targeted by the persistent Dam MTase was accounting for more than 30,000 methylated sites. Two motifs (CAGNNNNNGTG and CACNNNNNCTG) are presumably targeted by the same MTase. This assumption is based on the number of each of these motifs found in the genome (both accounting for 2021 methylated sites) and because they are mapping at both strands of the same loci. They could therefore be considered as partner motifs. The last identified motif (AANNNCCGGGNNNNNGA), accounting for only 73 methylated sites, is defined as a new motif according to REBASE, since it has not been described in other bacterial strains up to now. Given its low objective score (Table 1), we cannot rule out that the prediction of this motif could be slightly inaccurate. Its fraction of methylation was, however, high enough (0.76) to be considered as a genuine motif in this analysis. All of the methylated nucleotides found in these four motifs were distributed across the genome, as shown in Figure 2. In addition, for easier access to the data, a dedicated webpage with a genome browser displaying the precise position of the methylated nucleotides was generated (access details can be found in the “Data Availability” section).

As we recently sequenced the genome of three strains belonging to another *Xenorhabdus* species, *X. kozodoii*, we analyzed their methylome and present the main results here. Sequencing yielded an average coverage of 167×, 246×, and 260× for the strains FR48, FR71, and FR74, respectively. It allowed the identification of a high number of DNA modification marks (n = 38,077, 42,539, and 38,440 for the strains FR48, FR71, and FR74, respectively) (Table 1). As for *X. nematophila*, the most predominant motif was GATC targeted by Dam, conserved in all three *X. kozodoii* strains and accounting for >30,000 sites in each genome. Besides GATC, two other motifs were found in two *X. kozodoii* strains: GACCC in FR71 and FR74, and the partner-motif CATCNNNNNNCTC/GAGNNNNNNGATG in FR48 and FR74. All other identified motifs were found only in one of the tested strains. Only one of the motifs among those identified in the three *X. kozodoii* strains is defined as a new motif according to the REBASE database (Table 1).

### 2.3. MTase Expression

**Figure 3:**
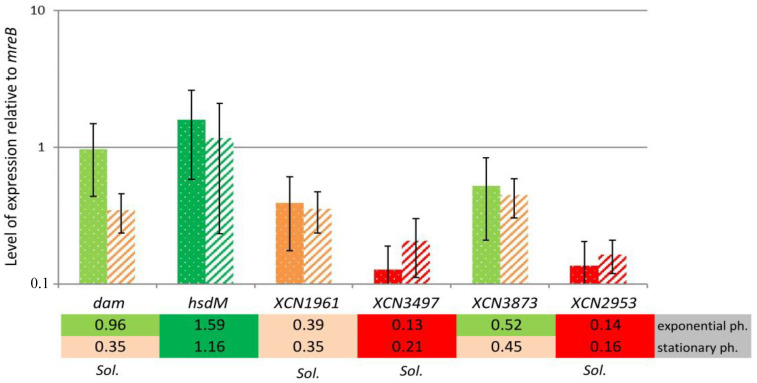
**Expression level of the 6 MTases-encoding genes in *X. nematophila* F1.** The expression ratio of each MTase gene relative to the *mreB* housekeeping gene is indicated. Measurements were performed on RNA extracted from 3 independent experiments during growth in exponential (dotted bars) or stationary (hatched bars) phase. Various colors represent various ranges of level of expression. Green, >1-fold the *mreB* gene; light green, >0.5-fold the *mreB* gene; orange, >0.3-fold the *mreB* gene; red, <0.3-fold the *mreB* gene. *sol.*: solitary MTase.

The expression level of the six MTase-encoding genes found in the *X. nematophila* F1 strain was analyzed by qRT-PCR relative to the housekeeping gene *mreB*, on mRNA extracted from cells grown in LB and harvested during exponential phase and stationary phase. Figure 3 shows that the six MTases could be split into three groups: (i) high level of expression: MTases encoded by a gene with a level of expression more than one-fold that of *mreB*; (ii) intermediate level of expression: MTase genes that were expressed at a lower but significant level (>0.5-fold the *mreB* level of expression) during exponential phase, while expressed to a lesser extent during stationary phase; (iii) low level of expression: MTase genes with a much lower level of expression when compared to *mreB* (less than 0.5-fold) regardless of the growth condition tested.

MTases associated with an RM system (*hsdM* = XNC3_v3_102 and putatively XNC3_v3_3873) were affiliated to the group with a high or intermediate level of expression, respectively. Among the solitary MTase-encoding genes, only *dam* could be assigned to the group with an intermediate level of expression (Figure 3). Finally, given their very low expression levels, the three other solitary MTase-encoding genes (XNC3_v3_1961, XNC3_v3_3497, and XNC3_v3_2953) were affiliated to the group with a low level of expression and could be assumed to be inactive. Therefore, they presumably do not significantly contribute to the genome methylation pattern in *X. nematophila* F1. Altogether, only three out of six MTases found in this strain seem to be active. This is in agreement with the methylome results that allowed the identification of four distinct motifs, with two of them being partner-motifs targeted by a single MTase (Table 1).

### 2.4. Unmethylated GATC Sites Are More Frequently Associated with Intergenic Regions

We investigated the location of unmethylated GATC motifs in *X. nematophila* F1, since such unmethylated sites could represent novel regulatory sites in the genome, where a competition between Dam and a DNA binding protein may occur [5,31].

**Figure 4:**
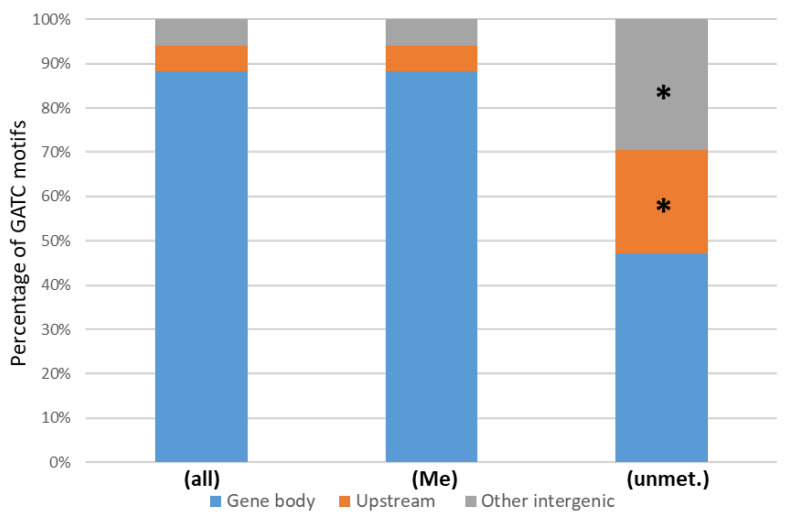
**Location of unmethylated GATC motifs**. Proportion of GATC motifs located in gene body (blue), in putative promoter (i.e., <100 bp upstream from a start codon) (orange), or in intergenic region (grey). (all), all motifs found in the genome; (Me), methylated motifs; (unmet.), unmethylated motifs. Asterisks indicate that the proportion of motifs located in upstream region vs. gene body is significantly different from the proportion observed in the (all) condition (*p* < 0.01, Fisher’s exact test).

The number of unmethylated GATC motifs are rare in *X. nematophila* (n = 77, see genome browser; access details can be found in the “Data Availability” section), according to the high fraction of modifications marks identified. The location of the GATC motif-associated methylation marks was determined relative to the position of neighboring ORFs: either in a putative promoter region (i.e., <100 bp upstream from a start codon), intragenic (inside an ORF), or in other intergenic regions (i.e., >100 bp from a start codon, or downstream of an ORF). The fraction of GATC motifs with modification marks mapping to a putative promoter region, as well as the fraction of motifs without modification marks, was calculated. These fractions were compared to the fraction of the corresponding motifs mapping to putative promoter regions found in the genome (Figure 4). Results showed that the fraction of unmethylated GATC motif located in putative promoter regions was significantly higher than that observed elsewhere in the genome (*p* < 0.005, Fisher’s exact test).

### 2.5. Phenotypes Associated with Dam Overexpression

The role of *X. nematophila* F1 *dam* gene was investigated by using a strain overexpressing *dam*. In *X. nematophila*, genes placed under the control of the P*_lac_* promoter are constitutively expressed [32]. Therefore, the additional copy of the *dam* gene caused by the presence of pBB-Dam plasmid, together with constitutive expression of the strong P*_lac_* promoter, are presumably causing a *dam* overexpression in *X. nematophila*, as previously observed in *Photorhabdus* [26]. In order to confirm this hypothesis, we quantified the level of expression of the *dam* gene. RT-qPCR experiments showed an average of 14.5-fold induction of expression of *dam* in *X. nematophila* harboring pBB-Dam when compared to the control strain (i.e., harboring a pBBR1MCS-5 empty plasmid) during exponential phase, and a 28.5-fold increased expression during stationary phase.

**Figure 5:**
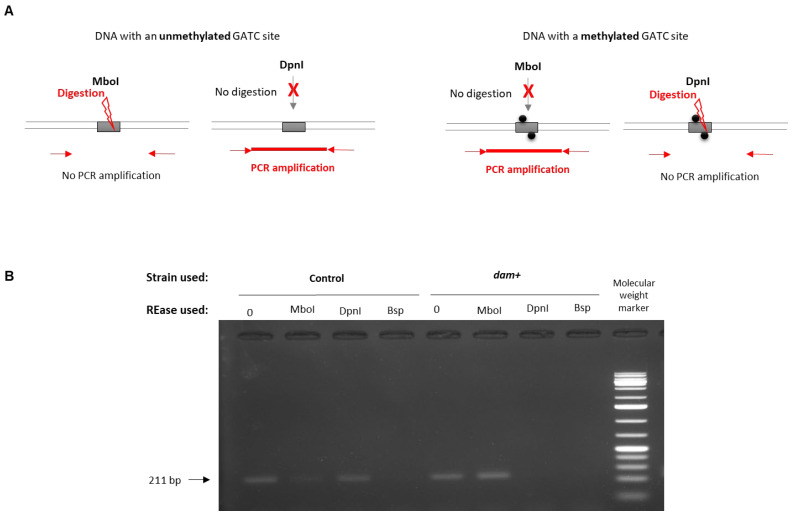
**Methylation-sensitive restriction enzyme (MSRE)-PCR analysis.** (**A**) Theoretical representation of a DNA region with a methylated (grey box with black circles) or unmethylated (grey box) GATC site, treated by MboI or DpnI restriction enzymes, and followed by PCR amplification (for which primers are represented by red arrows). (**B**) PCR amplification of a *X. nematophila* F1 locus (in the vicinity of XNC3v3_2082) harboring two unmethylated GATC sites was performed using DNA extracted from *X. nematophila* overexpressing *dam* strain (*dam+)* or from the control strain (carrying the pBBR1-MCS5 empty vector) after incubation with various restriction endonucleases: MboI (digests unmethylated GATC), DpnI (digests methylated GATC), or Bsp143I (digests GATC sites, whatever the methylation state). A positive-control PCR with undigested DNA (labelled as “0”) was performed. A lack of detection of a 211 bp amplicon revealed that digestion occurred.

In order to determine if the DNA-methylation pattern in *X. nematophila* was modified by the *dam* overexpression, an MSRE (methylation-sensitive restriction enzyme)-PCR approach was used on a locus harboring GATC sites that were identified as unmethylated in the *X. nematophila* WT strain (see genome browser). Results are presented in Figure 5. Detection of an amplicon revealed that no digestion occurred: this was observed for DpnI treatment in the control strain, confirming that the GATC sites of this region were unmethylated, and for MboI treatment in the *dam*-overexpressing strain, showing that the GATC sites were methylated. In parallel, no (or a weak) amplification was observed for MboI treatment in the control strain, confirming that the GATC sites of this region were unmethylated. Similarly, for DpnI treatment in the *dam*-overexpressing strain, the absence of amplification revealed the presence of methylated GATC sites. Altogether, these results indicate that the overexpression of the *dam* gene modifies the methylation pattern of the *X. nematophila* DNA.

Several phenotypes were assessed to compare the *dam*-overexpressing strain to the control strain. Growth in LB was monitored with an automated turbidimetric system, and the growth curves of both strains overlapped with the same shape: their slopes were similar during the exponential phase and they reached the same maximum OD during stationary phase (Supplementary Materials Figure S1). In addition, no significant difference was observed between *X. nematophila* harboring pBB-Dam when compared to the control *X. nematophila* strain, for bromothymol blue adsorption on NBTA, antibiotic production, and lipase activities (Table 2). Altogether, these findings indicate that these *X. nematophila* phenotypes are not altered by *dam* overexpression.

**Figure 6:**
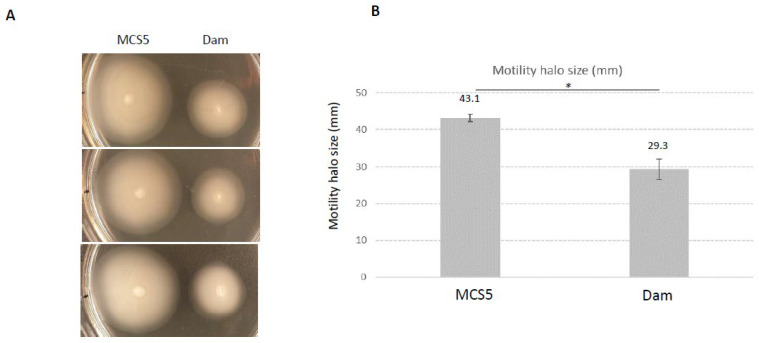
**Swimming motility of *X. nematophila* F1 overexpressing *dam* gene (Dam) and control (MCS5).** (**A**) Swimming halos were observed on low agar LB medium inoculated with 5 µL of exponentially growing cells (3 independent biological replicates are shown). (**B**) Halo size of motility of each strain measured after 24 h of incubation. * Difference between the two strains is significant (*p* < 0.05, Student’s *t*-test).

**Figure 7:**
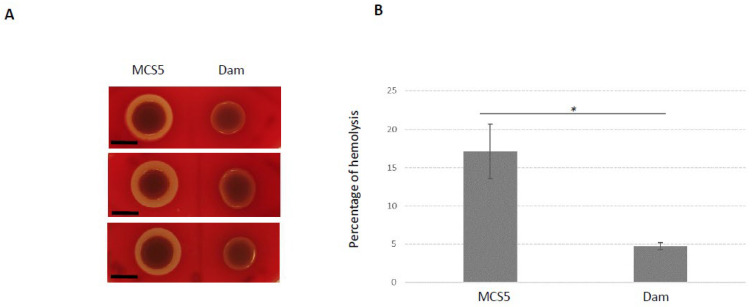
**Hemolytic activity produced by *X. nematophila* F1 overexpressing *dam* gene (Dam) and control (MCS5).** (**A**) Halos of hemolysis were observed on sheep blood agar medium inoculated with 5 µL of exponentially growing cells (3 independent biological replicates are shown; Black bars, 5 mm). (**B**) Liquid hemolytic assay: the released hemoglobin following lysis of sheep red blood cells was measured for each strain from cells grown 8 h in LB. * Difference between the two strains is significant (*p* < 0.05, Student’s *t*-test).

**Figure 8:**
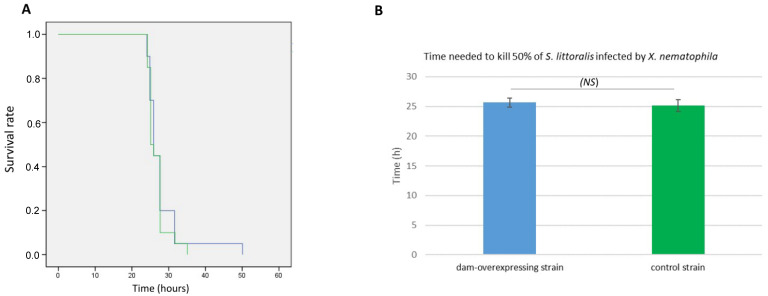
**Infection of *Spodoptera littoralis* larvae by *X. nematophila* overexpressing *dam* and the control strain.** (**A**) Proportion of survival of *S. littoralis* after injection of 10^4^ CFU of *X. nematophila* overexpressing *dam* (blue) or carrying the vector control (pBBR1-MCS5, green). Presented results are from one experiment (with 20 insect larvae infected for each bacterial strain), representative of three independent experiments. (**B**) The histograms represent the mean values (± standard error to the mean) of the time needed to kill 50% of infected larvae (LT_50_) monitored from 3 independent experiments. The LT_50_ were not significantly different between the two strains (NS, *p* > 0.05, Student’s *t*-test).

In contrast, two phenotypes previously described as associated with virulence in *X. nematophila* seemed impaired by *dam* overexpression: motility and hemolysis (Table 2). We therefore performed additional experiments to characterize these two phenotypes more in depth. Results presented in Figure 6 show that motility was significantly reduced in the *dam* overexpressing strain compared to the control strain. Similarly, the hemolytic activity was also significantly impaired in this strain (Figure 7). In light of these observations, the insect virulence of the *X. nematophila* strain overexpressing *dam* was compared to that of the control strain. It was assessed by injection of *X. nematophila* in *Spodoptera littoralis* larvae. Both strains were highly pathogenic, being able to cause death of all injected larvae in <48 h. The time needed to kill 50% of infected larvae (LT_50_) was below 30 h and was not significantly different between the two strains in three independent experiments (Figure 8). This suggests that *dam* overexpression did not modify the virulence properties of *X. nematophila*.

### 2.6. Flagellar Genes Are Downregulated in the X. nematophila dam-Overexpressing Strain

**Figure 9:**
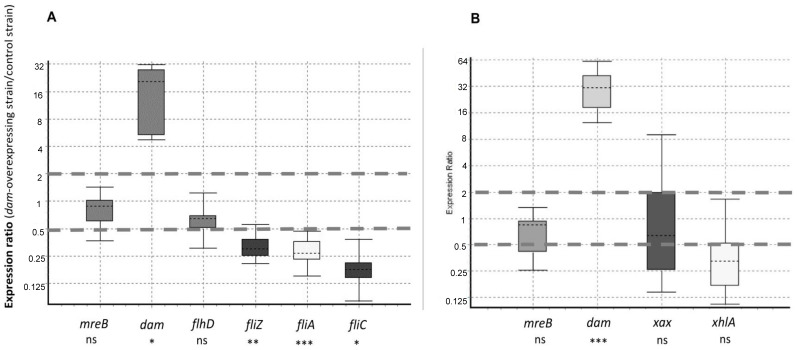
**Relative expression of 9 selected genes in the *X. nematophila dam*-overexpressing strain.** qRT-PCR was carried out with total RNA extracted from cells of the *dam*-overexpressing strain and of the control strain harvested during exponential (**A**) or stationary (**B**) phase of growth. Box plot representing the expression ratio between the two strains is shown for each tested gene, with *recA* used as a reference gene (see Materials and Methods section for details). Results are from 3 independent cultures for each strain. Expression ratios between 0.5 and 2 are flanked by dotted gray lines. The level of expression between the 2 strains was different at *p* < 0.05 (*), at *p* < 0.01 (**), at *p* < 0.001 (***), or was not significantly different (ns, *p* > 0.05), depending on the tested genes.

We wondered if the impaired hemolysis and the reduced motility observed for the *X. nematophila* strain overexpressing *dam* were associated with changes in gene expression. Genes related to these two phenotypes were therefore selected and their level of expression was quantified by qRT-PCR in the *X. nematophila* strain overexpressing *dam* in comparison to the control strain. As mentioned above, the *dam* gene was upregulated 14.5- to 28.5-fold depending on the growth phase considered (Figure 9). Results also showed that three of the four tested flagellar genes were significantly downregulated in the Dam-overexpressing strain during the exponential phase (Figure 9), suggesting that the motility phenotype was impacted at the transcriptional level. The expression of *flhD*, encoding the master flagellar regulator, was the only flagellar gene showing no significant change in expression in the Dam-overexpressing strain. In addition, the level of expression of two genes (*xhlA* and *xaxA*) known to be involved in hemolysis of sheep red-blood cells in *X. nematophila* was investigated during stationary phase of growth (i.e., a condition when hemolysis activity is detected [33,34]), and qRT-PCR experiments revealed that the level of expression of these genes was not significantly different between the two strains (Figure 9).

## 3. Discussion

Almost half a century ago, DNA methylation was proposed to contribute to epigenetic regulation in multicellular eukaryotes [35]. At the same time, DNA methylation was also studied in prokaryotes for its involvement in restriction-modification systems [36]. DNA methylation was later shown to be associated with epigenetic regulation in prokaryotes as well [37,38]. This phenomenon has multiple functions in the cell, ranging from genome maintenance (i.e., DNA mismatch repair, initiation of chromosome replication) to regulation of gene expression as an epigenetic mechanism [2,6]. Perturbations in the DNA methylation pattern have been associated with modification of diverse phenotypes in bacteria [13,28], illustrating the fact that studying the enzymes responsible for DNA-methylation in a broad context can provide new information about their importance in the life cycle of bacteria.

In the present study, the distribution of MTases encoding genes was shown to vary significantly among the *Xenorhabdus* genus, ranging from 3 to 18 putative MTases depending on the strains studied. Such a diversity in MTases distribution among a single bacterial genus has already been observed elsewhere, in both Gram-negative [30,39,40] and Gram-positive bacteria [5,41]. Most of these genes were found in a limited number of strains of the *Xenorhabdus* genus, suggesting that they originate from horizontal gene transfers. The Dam MTase was the only one always conserved in *Xenorhabdus*, and orthologs of this persistent MTase are also found in numerous Gram-negative bacteria [6]. The investigation of the level of expression of the MTases encoding genes found in *X. nematophila* revealed that three out of six MTases found in the studied strain seemed to be weakly transcribed, compared to the level of expression of a housekeeping gene. This is in agreement with similar observations that were made elsewhere [30,40] and with the fact that bacterial methylome analyses usually identify much fewer methylated motifs than the putative number of MTase encoding genes for a given strain [5]. For some of the MTases encoding genes that were significantly expressed in *X. nematophila*, slight variations could be observed when comparing the level of expression between exponential phase and stationary phase of growth. Bacterial MTases are very efficient enzymes as long as they can reach their DNA targets [42], and another study suggested that a limited increase or decrease in the level of expression of MTases encoding genes did not significantly contribute to the genome methylation pattern [30]. We can then assume that this is similar in *Xenorhabdus*.

Here, PacBio sequencing performed on two different species of the genus *Xenorhabdus* allowed detecting numerous methylation marks distributed all over their genomes and mapping in specific motifs. This methylome analysis allowed the identification of new methylated motifs, as well as their occurrence in the respective genomes. As already observed for many other Gram-negative bacteria, the most prevalent methylation motif was GATC, which is methylated by Dam [5,30,40]. Besides the GATC motif, the methylated motifs identified in *X. nematophila* F1 were different when compared to those previously reported for the ATCC19061 strain [5]. This is in agreement with the varying MTase repertoire identified in their respective genomes, as described in the present study, and similar observation could be made for the three *X. kozodoii* strains studied here. Altogether, these results confirm that the MTases repertoire, and consequently the identified methylated motifs, can vary significantly between strains of a given species [39,41].

Methylation by Dam MTase is very efficient in *Xenorhabdus*, causing the DNA methylation of more than 99% of the GATC motifs in the investigated genomes, a rate of methylation similar to what has already been described in the closely related genus *Photorhabdus*, as well as in other Gram-negative bacteria such as *E. coli* or *Salmonella* [30,43,44]. As observed for other bacteria, despite this high level of methylation, a few GATC motifs that were unmethylated in the tested conditions could be identified [30,43,45,46]. The observed enrichment of these unmethylated motifs in putative promoter regions suggests the existence of factors, such as DNA-binding proteins, that hinder these particular GATC sites and that may be responsible for epigenetic regulations [2,31]. Although the coupling of differentially methylated and differentially transcribed genes is not as frequent as one may expect [31,43,45] it can still be significant enough to allow the detection of many genes likely to be epigenetically regulated [46]. The literature has thus reported several regulators able to cause epigenetic regulation due to a differential affinity for DNA, depending on the DNA methylation state. The main regulators described so far are OxyR, Lrp, and Fur [47,48,49,50]. Such differential methylation pattern of the GATC sites found in promoters causes a differential expression of several genes, often encoding virulence factors. Major examples are *pap*, *agn43*, and *sci1* in *E. coli* [37,49,50], but also *gtr* or *opvAB* in *Salmonella* [44,51]. Regarding Lrp in *X. nematophila*, a virulence attenuation in insects was shown to be associated with a mutation in the *lrp* gene [52], and this regulator presumably contributes to bacterial motility since it was shown to positively regulate the expression of the master flagellar regulator FlhD [53]. However, none of the unmethylated GATC sites identified in the present study mapped in the vicinity of flagellar encoding genes, suggesting that the motility phenotype in *Xenorhabdus* does not involve direct epigenetic regulation associated with DNA methylation.

A powerful tool to identify phenotypes associated with DNA methylation in bacteria is to modify the level of expression of MTases, consequently modifying the DNA methylation pattern [13]. Studying knockout mutant of MTases is a drastic way to modify the DNA-methylation pattern. This often leads to strong modification of phenotypes [2,27]. Because Dam MTase is widely distributed in *Enterobacteriaceae*, its role has been extensively studied. It displays pleiotropic roles in relation to its typical DNA-methylation activity [6], and in a few bacterial species, an essential function for Dam in cell viability was also proposed [19,23]. Moreover, a critical role of Dam has been reported in several bacterial species during bacterial–host interactions [28]. This includes major mammalian pathogens as described above in the introduction [16,17,20,21,23,24,54,55,56]. Another subtler manner to identify phenotypes associated with DNA methylation in bacteria is the use of MTase-overexpressing strains. Such an approach has also unveiled the identification of some phenotypes important for the life cycle of several bacteria [19,22,26,56,57]. However, despite the major phenotypes investigated, in most of the abovementioned studies, no specific epigenetic regulation mechanism was described. DNA-methylation and transcriptional regulation were also shown to be largely decoupled in *Salmonella* [45], suggesting that identification of mechanisms of epigenetics regulations can be tricky in bacteria. In the present work, Dam overexpression did not reveal an important role for this MTase in the virulence properties of *X. nematophila* against insect larvae. Several other phenotypes were also found unmodified by the *dam* overexpression during growth in standard conditions. However, two major phenotypes were impaired when compared to the control strain: motility and hemolysis were significantly reduced in the *X. nematophila* strain overexpressing *dam*. We showed that Dam overexpression is associated with a modification of the DNA methylation pattern in *X. nematophila*, as also observed in the closely related bacterium of the genus *Photorhabdus* in similar conditions [30]. Methylome analysis in bacteria is only a first step towards identification of putative epigenetic regulations [46]. In the present study, none of the GATC motifs identified as unmethylated mapped near genes for which the function could be associated with the observed impaired phenotypes (see genome browser for details). This observation includes the flagellar encoding genes identified here as downregulated in the Dam-overexpressing strain. In addition, no significant change in expression of the tested genes encoding hemolysins was observed in the *dam*-overexpressing strain, despite an impaired hemolytic phenotype. Altogether, this suggests the existence of several distinct and indirect pathways between DNA methylation and the observed phenotypic changes. Although the specific mechanisms by which DNA methylation impairs these phenotypes remains unknown, our findings suggest that genome-wide alterations of methylation patterns in *X. nematophila* can significantly impact some important phenotypes. Interestingly, these two phenotypes were previously shown as being linked in *Xenorhabdus*, together with insect virulence [58,59]. Given the large repertoire of *Xenorhabdus* factors involved in bacterial–host interaction [53], several other genes are susceptible to contribute to virulence. In contrast to what was observed in *Xenorhabdus*, virulence property was significantly impaired by *dam* overexpression in *Photorhabdus* [26]. Taken together, these results reveal that DNA methylation pattern can contribute in different manners in cell physiology, even for closely related bacteria with similar life cycles.

In conclusion, although no direct epigenetic regulation was revealed by this study, it allowed the identification of new phenotypes associated with overexpression of an MTase and therefore confirms that such a strategy can be a powerful tool to investigate the role of DNA-MTases in bacteria.

## 4. Materials and Methods

### 4.1. Strains and Growth Conditions

The bacterial strains and plasmids used in this study are listed in Table 3. Two different species of the genus *Xenorhabdus* initially isolated from entomopathogenic nematodes were used in this study: in addition to the *X. nematophila* F1 laboratory model strain [60], we also used 3 different strains of *X. Kozodoii*, for which their complete genomes were sequenced in the course of this study (see Section 4.3). *E. coli* and *Xenorhabdus* cells were routinely grown in Luria broth (LB) medium with a 180 rpm agitation at 37 °C and 28 °C, respectively. As required, antibiotic concentrations used for bacterial selection were gentamycin (Gm) at 15 μg mL^−1^.

### 4.2. Distribution of DNA-Methyltransferases in Xenorhabdus by In Silico Analysis

The REBASE database [7] was used to identify the putative DNA-methyltransferases (MTase) in 12 genomes from 9 species of *Xenorhabdus*: *X. bovienii*, *X. doucetiae*, *X. griffae*, *X. hominickii*, *X. khoisanae*, *X. kozodoii*, *X. nematophila*, *X. poinarii*, and *X. szentirmaii*. For each identified MTase, the distribution of putative orthologs among other strains of *Xenorhabdus* or in other Gram-negative bacteria was performed on the OmicsBox platform (OmicsBox—Bioinformatics Made Easy, BioBam Bioinformatics, 3 March 2019, https://www.biobam.com/omicsbox, accessed on 1 September 2022) using Blast2Go software [64]. We used the BlastP default setting and retrieved the first 100 hits. Only proteins with >70% sequence identity were considered as orthologs. The phylogenetic tree presented in Figure 1 was built using the genome clustering tool of the MaGe Platform (https://mage.genoscope.cns.fr/, accessed on 1 September 2022). Briefly, the genomic similarity was estimated using Mash [65], a software that computes a distance between two genomes. This distance was correlated to the ANI as D ≈ 1-ANI. From all the pairwise distances of the genomes set, a tree was constructed dynamically using the neighbor-joining Javascript package. Genomes were grouped into the same cluster of species when the calculated pairwise distance was ≤0.06 (≈94% ANI). The MTases listed in Figure 1 were classified using the UPGMA distance method (Jaccard similarity coefficient) via the DendroUPGMA server (http://genomes.urv.cat/UPGMA/, accessed on 1 September 2022). For the *X. nematophila* F1 strain, MTases annotation is indicated.

### 4.3. Genome Sequencing and DNA Methylation Detection and Motifs Identification

Genomic DNA was extracted from bacteria grown in LB and harvested at an OD_540_ of 1.5 as follows. Bacterial cells corresponding to 2 mL of culture were washed in PBS and pellets were stored at −80 °C. To perform lysis, cells were resuspended in 200 µL of TSE-lysozyme for 15 min at 37 °C, followed by addition of 640 µL EDTA pH8 0.5 M and 160 µL SDS 10% and incubated 15 min at 60 °C. Lysates were incubated for 1 h at 56 °C after addition of 20 µL proteinase K (20 mg·mL^−1^), cooled on ice, and incubated 5 min at room temperature with 30 µL of RNAse A (20 mg·mL^−1^). Precipitation of contaminants was performed by addition of chilled 350 µL potassium acetate 5 M and a centrifugation step (10,000× *g* for 10 min at 4 °C). The genomic DNA was purified with magnetic beads (Sera-Mag Speed beads, Thermo-Scientific, Courtaboeuf, France) as previously described [30].

Since the *X. nematophila* and *X. kozodoii* genomes were sequenced on different platforms, the protocols used slightly differ between the two species, and are detailed below.

For *X. nematophila*, the DNA libraries were prepared according to PacBio guidelines as follows: 20 kb Template Preparation Using BluePippin Size-Selection System (15 kb size cutoff); shearing at 40 kb was performed using Megaruptor system (Diagenode); sizing at 17 kb was performed using BluePippin system (Sage). Libraries were sequenced on one PacBio SMRT cell at 0.25 nM with the Protocol OneCellPerWell (OCPW), P6C4 chemistry, and 360 min movies on a Pacific Biosciences RSII instrument (GeT-PlaGe, Toulouse, France).

The reads were assembled de novo, with the high-quality Hierarchical Genome Assembly Process (HGAP4) from SMRT Link 5.0.1, and produced one contig corresponding to the chromosome. The *X. nematophila* F1 genome can be found at https://mage.genoscope.cns.fr/microscope/mage/index.php (accessed on 1 September 2022). A second, manual step was performed using the unassembled reads to identify plasmids. All unassembled reads with a hit on the chromosome or included in a larger read were excluded, and the remaining reads were aligned against Swissprot bacteria with BLASTX to find proteins with an annotation corresponding to a plasmid. Finally, chromosome and plasmids were polished with Illumina single-end data with BWA 0.7.15-r1140 and Pilon v1.22 software.

For *X. kozodoii*, the sequencing was performed according to PacBio guidelines on a Pacific Biosciences Sequel I instrument (Gentyane, Clermont-Ferrand, France), using a multiplexing step of 5 samples for 1 SMRT-cell. *X. kozodoii* assemblies were made with HGAP4 (SMRT Link 5.1.0), then the origin of replication was placed using BLAST 2.7.1. Finally, the genomes were polished with Blasr and Arrow (SMRT Link 5.1.0).

For all 4 *Xenorhabdus* strains, DNA methylation was determined using the ds_modification_motif_analysis protocol within SMRT Link 5.1.0, which uses an in silico kinetic reference and a Welch’s-*t*-test-based kinetic score detection of modified base positions with parameters set as follows: subread/polymerase read length ≥ 500, polymerase read quality ≥ 80, and modification QV ≥ 30. A score of 30 for the modification QV is the default threshold for calling a position as modified, and corresponds to a *p*-value of 0.001. Homemade script was used to keep methylated bases for adenine or cytosine with score ≥ 30 and known motifs.

### 4.4. Nucleic Acid Manipulations

The extraction of plasmid DNA from *E. coli* was performed using the GenElute™HP Plasmid miniprep purification kit as recommended by the manufacturer (Sigma, Saint-Quentin-Fallavier, France). Chromosomal DNA was extracted from bacterial cells using the QIAamp DNA Mini kit (Qiagen, Courtaboeuf, France). Restriction enzymes and T4 DNA ligase were used as recommended by the manufacturer (New England Biolabs, Evry, France and Promega La Farlede, France, respectively). Oligonucleotide Primer sequence was designed using the Primer3 software (Untergasser et al., 2012). They were synthesized by IDT (Leuven, Belgium) and are listed in Table S1. PCR was performed in a T100 thermal cycler (Biorad, Marnes-la-Coquette, France) using the iProof high-fidelity DNA polymerase (Biorad). Amplified DNA fragments were purified using a PCR purification kit (Ozyme, St Cyr L Ecole, France) and separated on 0.7% agarose gels after digestion as previously described [66]. Digested DNA fragments were extracted from agarose gels with a centrifugal filter device (Ozyme). All constructions were confirmed by DNA sequencing (Eurofins Genomics, Nantes, France). Cloning the *X. nematophila dam* gene was performed as follows. The XNC3_v3_0322 gene was PCR amplified using two primers mapping upstream and downstream (Cp-dam0322-F and Cp-dam0322-RF, respectively, Table S1) the 804 bp ORF (open reading frame), using the following cycling conditions: 98 °C, 10 s; 56 °C, 30 s; 72 °C, 30 s for 35 cycles. The 905 bp long amplified DNA fragment was then digested according to the endonuclease sites introduced in the primers (EcoRI and BamHI), and was inserted between the corresponding sites of the low-copy plasmid pBBR1MCS-5 [63] downstream of the Plac promoter, as previously described [26], and transformed into XL1 blue MRF’ strain. The recombinant plasmid (pBB-dam) was introduced in the *E. coli* WM3064 donor strain by electroporation, and then transferred in *X. nematophila* by conjugative mating as previously described [58]. *X. nematophila* harboring the pBBR1MCS-5 empty plasmid was used as a control.

### 4.5. Methylation-Sensitive Restriction Enzyme (MSRE) PCR Analysis

Changes in DNA-methylation pattern by *dam* overexpression was tested by MSRE-PCR on a locus (in the vicinity of XNC3v3_2082) that was chosen because it harbors 2 unmethylated GATC sites in *X. nematophila* F1 wild-type strain. Digestion using methylation-sensitive restriction endonucleases was followed by PCR amplification, based on a previous protocol [67] and described below. First, 1 µg of genomic DNA from *X. nematophila* F1 strains, either overexpressing *dam* or its control strain (carrying the pBBR1-MCS5 empty vector), was diluted to 50 ng/µL, and 500 ng were digested by EcoRI for 2 h at 37 °C in order to generate numerous linear fragments, followed by an enzyme inactivation step (20 min at 65 °C). Ten ng of DNA were then digested by 5 U of MboI, a restriction enzyme that digests only unmethylated GATC sites, or DpnI, a restriction enzyme that digests only methylated GATC sites. Positive and negative control reactions were performed similarly using either 5 U of Bsp143I (which digests GATC sites, whatever their methylation state) or water, respectively. PCR amplification was performed on 1 ng DNA (25 s, 94 °C; 25 s, 57 °C; 20 s, 72 °C for 28 cycles) using MSRE-2082-F and MSRE-2082-R primers (Table S1) and then loaded onto a 1.5% agarose gel. Detection of an amplicon revealed that no digestion occurred (i.e., the GATC sites of this region were methylated for MboI treatment and unmethylated for DpnI treatment), while no amplification revealed that the region was digested (i.e., at least one of the GATC sites of this region was unmethylated for MboI treatment, and at least one of the GATC sites of this region was methylated for DpnI treatment).

### 4.6. Phenotype Analysis of X. nematophila Overexpressing Dam

Growth was monitored with a TECAN automated turbidimetric system (Infinite M200 TECAN, Lyon, France). Bromothymol blue adsorption was determined after growth on NBTA (nutrient agar supplemented with 25 mg of bromothymol blue and 40 mg of triphenyltetrazolium chloride per liter). It allows the identification of variant forms [68]. Antibiotic production was assessed by measuring antibacterial activity against *Micrococcus luteus* (Table 3). Hemolysis was determined by the observation of a clearing surrounding bacteria grown on standard sheep blood agar plates, and quantified by a liquid hemolytic assay as previously described [34,69]. Lipase activities on Tween 20, 40, 60, 80, and 85 were also assessed as previously described [68].

### 4.7. Insect Virulence Assay

The virulence-related properties of *dam* overexpression were assessed by comparing the killing effect of *X. nematophila* transconjugants harboring either the pBB-Dam or the pBBR1MCS-5 empty plasmid during infection in the common cutworm *Spodoptera littoralis* as previously described [33]. Briefly, 20 μL of exponentially growing bacteria (DO_540nm_ = 0.3) diluted in LB, corresponding to about 1 × 10^4^ CFU, were injected into the hemolymph of 20 sixth-instar larvae of *S. littoralis* reared on an artificial diet. Three independent pathogenicity assays were performed for each bacterial strain. Insect larvae were then individually incubated at 23 °C. Insect death was monitored over time. The time for killing 50% of the insect larvae (LT_50_) was determined for each of these three independent experiments and the mean value was calculated for each strain. Student’s *t*-test was performed to compare the LT_50_ between the two strains.

### 4.8. RNA Preparation and RT-qPCR Analysis

Total RNA extraction was performed on cells harvested at OD_540nm_ = 0.5 or 1.5 (exponential or stationary phase of growth, respectively), from three independent cultures for each strain, using RNeasy miniprep Kit (Qiagen), according to the manufacturer’s instructions. An additional incubation step with DNase I (Qiagen) was performed when required. The quantity and quality of RNA were assessed with an Agilent 2100 Bioanalyzer (Les Ulis, France) with the RNA 6000 Nano LabChip kit. Lack of DNA contamination was controlled by carrying out a PCR on each RNA preparation.

Quantitative reverse transcription-PCR (RT-qPCR) was carried out as previously described [70]. Briefly, the SuperScript II reverse transcriptase (Invitrogen) was used on 0.5 μg of total RNA with random hexamers (100 ng/μL; Promega). qPCR analyses were performed in 1.5 μL using SensiFAST SYBR No-ROX kit (Bioline), 0.5 μL of cDNA synthesis mixture (diluted 1:20), and 1 μM specific primers for the studied genes (Table S1). The enzyme was activated by heating for 2 min at 95 °C. All qPCRs were performed in three technical replicates, with 45 cycles of 95 °C for 5 s and 61 °C for 30 s, and were monitored in the LightCycler 480 system (Roche). Melting curves were analyzed for each reaction, and each curve contained a single peak. For standard curves, the amounts of PCR products generated were determined with serially diluted genomic DNA from *X. nematophila*. To display the MTase-encoding genes level of expression in the WT strain of *X. nematophila*, the relative transcript level of each tested gene versus the *mreB* housekeeping gene, as calculated with LightCycler 480 software (Roche), is represented as histograms (Figure 3). The data shown are the medians of experimental triplicates, and error bars represent the statistical standard deviations. To display the comparison in the level of expression of 9 selected genes between the *dam*-overexpressing strain and the control strain (Figure 9), the data were analyzed with the REST software 2009 [71] using the pairwise fixed randomization test with 5000 permutations. Data are presented as a ratio with respect to the reference housekeeping gene *recA*, as previously described [70].

## Figures and Tables

**Table 1:** Methylated motifs detected by SMRT sequencing in *Xenorhabdus*.

Motif ^1^	Fraction	nDetected	nGenome	Mean Score	Mean Ipd Ratio	Mean Coverage	Objective Score
**Motifs in *X. nematophila* F1**							
GATC	0.998	31,731	31,808	95.6	5.52	56.9	3,028,101
CAGNNNNNGTG/	1.000	2021	2022	90.9	6.36	56.4	183,562
CACNNNNNCTG	1.000	2021	2022	87.5	5.32	56.7	176,691
AANNNCCGGGNNNNNGA ^2^	0.760	73	96	49.7	3.49	49.7	2838
**Motifs in *X. kozodoii* FR48**							
GATC	0.996	33,246	33,380	124.2	7.41	77.5	4,114,426
TTCANNNNNNGTG/	1.000	675	675	105.3	6.98	76.7	71,110
CACNNNNNNTGAA ^2^	1.000	675	675	92.7	5.74	76.0	61,087
CATCNNNNNNCTC/	0.991	425	429	111.2	7.08	74.3	46,883
GAGNNNNNNGATG	0.981	421	429	99.2	6.47	74.4	41,075
**Motifs in *X. kozodoii* FR71**							
GATC	0.997	33,373	33,460	176.4	7.43	115.0	5,874,492
GGATG	0.691	6281	9094	53.8	2.84	118.4	242,171
GACCC	0.936	2885	3082	61.4	3.25	116.9	166,942
**Motifs in *X. kozodoii* FR74**							
GATC	0.998	33,548	33,600	185.1	7.48	120.0	6,202,691
GACCC	0.953	2813	2953	64.4	3.22	124.7	173,373
TTCANNNNNNNGTG/	0.998	624	625	155.6	7.02	120.9	96,959
CACNNNNNNNTGAA ^2^	1.000	625	625	138.4	5.81	119.7	79,103
CATCNNNNNNCTC/	0.993	416	419	169.0	7.18	117.1	69,857
GAGNNNNNNGATG	0.988	414	419	147.2	6.48	117.0	60,273

^1^ Methylated base is underlined (m6A or m4C), and partner-motifs are indicated by /. ^2^ Methylated motif not described yet in REBASE.

**Table 2:** Phenotypes of *X. nematophila* F1 overexpressing *dam* gene (pBB-dam) and control (pBBMCS-5).

Strain	Tested Phenotypes ^a^								
	Btb Adsorption ^b^	Antibiotic Production ^c^	Sheep Blood Hemolysis ^d^	Motility ^e^	Lipolysis of ^f^				
					Tween 20	Tween 40	Tween 60	Tween 80	Tween 85
*X. nematophila* F1 WT	B	+	+	++	+	+	+	+	-
*X. nematophila* F1+pBBMCS-5	B	+	+	++	+	+	+	+	-
*X. nematophila* F1+pBB-dam	B	+	w	+	+	+	+	+	-

^a^ All plates were incubated for 2 days at 28 °C before assays were interpreted, unless otherwise indicated. Routinely tested phenotypes on the WT strain are indicated for comparison. ^b^ Btb, bromothymol blue; B, blue colonies on NBTA medium. ^c^ +, Halo size (>20 mm) of growth inhibition of *Micrococcus luteus*. ^d^ +, clear halo of hemolysis; w, reduced size halo of hemolysis (see Figure 2). ^e^ ++, Large spreading area (halo size > 40 mm); +, reduced spreading area (halo size < 30 mm) after 24 h of incubation. ^f^ +, Halo of precipitation; -, no halo of precipitation.

**Table 3:** Strains and plasmids used in this work.

Strain or Plasmid	Relevant Genotype and Characteristics	Reference or Source
**Strains**		
*Xenorhabdus nematophila* F1	Wild type	[60]
*Xenorhabdus kozodoii* FR48	Wild type	Laboratory collection
*Xenorhabdus kozodoii* FR71	Wild type	Laboratory collection
*Xenorhabdus kozodoii* FR74	Wild type	Laboratory collection
*Escherichia coli WM3064*	*thrB1004 pro thi rpsl hsdS lacZΔM15 RP4-1360Δ(araBAD)567 ΔdapA1341::[erm pir (wt)]*	[61]
*E. coli* MG1655	Wild type	[62]
*Micrococcus luteus*	Wild type	Pasteur Institute Culture collection, Paris, France
**Plasmids**		
pBBR1MCS-5	Cloning vector, Gm^R^	[63]
pBB-Dam	905 pb PCR fragment (*dam* gene) inserted between EcoRI and BamHI site of pBBR1_MCS5 plasmid	This study

## Data Availability

The datasets generated and analyzed during the current study are available as follows: EBI Genomic data (genomes, reads, annotations) are deposited under the project code PRJEB55110 (https://www.ncbi.nlm.nih.gov/bioproject/PRJEB55110 (which will be publicly released soon). Epigenomic data are available at: https://doi.org/10.57745/VLYY4R (for XnF1 strain), https://doi.org/10.57745/IW6GGB (for XkFR48 strain), https://doi.org/10.57745/OXGHDO (for XkFR71 strain), https://doi.org/10.57745/FVW8SY (for XkFR74 strain). A browser has also been generated to easily explore the detailed methylomic data of XnF1 strain: https://doi.org/10.25794/YW7M-SQ69.

## Supplementary Materials

The following supporting information can be downloaded at: https://www.mdpi.com/article/10.3390/ijms231911981/s1.

## References

[1] Lu S.C. (2000). S-Adenosylmethionine. Int. J. Biochem. Cell Biol..

[2] Sanchez-Romero M.A., Casadesus J. (2020). The bacterial epigenome. Nat. Rev. Microbiol..

[3] Billen D. (1968). Methylation of the bacterial chromosome: An event at the “replication point”?. J. Mol. Biol..

[4] Anton B.P., Roberts R.J. (2021). Beyond Restriction Modification: Epigenomic Roles of DNA Methylation in Prokaryotes. Annu. Rev. Microbiol..

[5] Blow M.J., Clark T.A., Daum C.G., Deutschbauer A.M., Fomenkov A., Fries R., Froula J., Kang D.D., Malmstrom R.R., Morgan R.D. (2016). The Epigenomic Landscape of Prokaryotes. PLoS Genet..

[6] Marinus M.G., Lobner-Olesen A. (2014). DNA Methylation. EcoSal Plus.

[7] Roberts R.J., Vincze T., Posfai J., Macelis D. (2015). REBASE--a database for DNA restriction and modification: Enzymes, genes and genomes. Nucleic Acids Res..

[8] Oliveira P.H., Touchon M., Rocha E.P. (2014). The interplay of restriction-modification systems with mobile genetic elements and their prokaryotic hosts. Nucleic Acids Res..

[9] Lobner-Olesen A., Skovgaard O., Marinus M.G. (2005). Dam methylation: Coordinating cellular processes. Curr. Opin. Microbiol..

[10] Casadesus J. (2016). Bacterial DNA Methylation and Methylomes. Adv. Exp. Med. Biol..

[11] Casadesus J., Low D. (2006). Epigenetic gene regulation in the bacterial world. Microbiol. Mol. Biol. Rev..

[12] Casadesus J., Low D.A. (2013). Programmed heterogeneity: Epigenetic mechanisms in bacteria. J. Biol. Chem..

[13] Low D.A., Casadesus J. (2008). Clocks and switches: Bacterial gene regulation by DNA adenine methylation. Curr. Opin. Microbiol..

[14] Balaban N.Q., Merrin J., Chait R., Kowalik L., Leibler S. (2004). Bacterial persistence as a phenotypic switch. Science.

[15] Weigel W.A., Dersch P. (2018). Phenotypic heterogeneity: A bacterial virulence strategy. Microbes Infect..

[16] Garcia-Del Portillo F., Pucciarelli M.G., Casadesus J. (1999). DNA adenine methylase mutants of *Salmonella* typhimurium show defects in protein secretion, cell invasion, and M cell cytotoxicity. Proc. Natl. Acad. Sci. USA.

[17] Heithoff D.M., Sinsheimer R.L., Low D.A., Mahan M.J. (1999). An essential role for DNA adenine methylation in bacterial virulence. Science.

[18] Wu H., Lippmann J.E., Oza J.P., Zeng M., Fives-Taylor P., Reich N.O. (2006). Inactivation of DNA adenine methyltransferase alters virulence factors in *Actinobacillus actinomycetemcomitans*. Oral Microbiol. Immunol..

[19] Erova T.E., Fadl A.A., Sha J., Khajanchi B.K., Pillai L.L., Kozlova E.V., Chopra A.K. (2006). Mutations within the catalytic motif of DNA adenine methyltransferase (Dam) of *Aeromonas hydrophila* cause the virulence of the Dam-overproducing strain to revert to that of the wild-type phenotype. Infect. Immun..

[20] Watson M.E., Jarisch J., Smith A.L. (2004). Inactivation of deoxyadenosine methyltransferase (*dam*) attenuates *Haemophilus influenzae* virulence. Mol. Microbiol..

[21] Mehling J.S., Lavender H., Clegg S. (2007). A Dam methylation mutant of *Klebsiella pneumoniae* is partially attenuated. FEMS Microbiol. Lett..

[22] Chen L., Paulsen D.B., Scruggs D.W., Banes M.M., Reeks B.Y., Lawrence M.L. (2003). Alteration of DNA adenine methylase (Dam) activity in *Pasteurella multocida* causes increased spontaneous mutation frequency and attenuation in mice. Microbiology.

[23] Julio S.M., Heithoff D.M., Provenzano D., Klose K.E., Sinsheimer R.L., Low D.A., Mahan M.J. (2001). DNA adenine methylase is essential for viability and plays a role in the pathogenesis of *Yersinia pseudotuberculosis* and *Vibrio cholerae*. Infect. Immun..

[24] Robinson V.L., Oyston P.C., Titball R.W. (2005). A *dam* mutant of *Yersinia pestis* is attenuated and induces protection against plague. FEMS Microbiol. Lett..

[25] Demarre G., Chattoraj D.K. (2010). DNA adenine methylation is required to replicate both *Vibrio cholerae* chromosomes once per cell cycle. PLoS Genet..

[26] Payelleville A., Lanois A., Gislard M., Dubois E., Roche D., Cruveiller S., Givaudan A., Brillard J. (2017). DNA Adenine Methyltransferase (Dam) Overexpression Impairs *Photorhabdus luminescens* Motility and Virulence. Front. Microbiol..

[27] Atack J.M., Tan A., Bakaletz L.O., Jennings M.P., Seib K.L. (2018). Phasevarions of Bacterial Pathogens: Methylomics Sheds New Light on Old Enemies. Trends Microbiol..

[28] Heusipp G., Falker S., Schmidt M.A. (2007). DNA adenine methylation and bacterial pathogenesis. Int. J. Med. Microbiol..

[29] Akhurst R.J., Boemare N.E. (1988). A numerical taxonomic study of the genus Xenorhabdus (Enterobacteriaceae) and proposed elevation of the subspecies of X. nematophilus to species. J. Gen. Microbiol..

[30] Payelleville A., Legrand L., Ogier J.C., Roques C., Roulet A., Bouchez O., Mouammine A., Givaudan A., Brillard J. (2018). The complete methylome of an entomopathogenic bacterium reveals the existence of loci with unmethylated Adenines. Sci. Rep..

[31] Payelleville A., Brillard J. (2021). Novel Identification of Bacterial Epigenetic Regulations Would Benefit From a Better Exploitation of Methylomic Data. Front. Microbiol..

[32] Lanois A., Pages S., Bourot S., Canoy A.S., Givaudan A., Gaudriault S. (2011). Transcriptional analysis of a *Photorhabdus* sp. variant reveals transcriptional control of phenotypic variation and multifactorial pathogenicity in insects. Appl. Environ. Microbiol..

[33] Brillard J., Duchaud E., Boemare N., Kunst F., Givaudan A. (2002). The PhlA hemolysin from the entomopathogenic bacterium *Photorhabdus luminescens* belongs to the two-partner secretion family of hemolysins. J. Bacteriol..

[34] Brillard J., Ribeiro C., Boemare N., Brehelin M., Givaudan A. (2001). Two distinct hemolytic activities in Xenorhabdus nematophila are active against immunocompetent insect cells. Appl. Environ. Microbiol..

[35] Holliday R., Pugh J.E. (1975). DNA modification mechanisms and gene activity during development. Science.

[36] Colson C., Van Pel A. (1974). DNA restriction and modification systems in *Salmonella*. I. SA and SB, two *Salmonella* typhimurium systems determined by genes with a chromosomal location comparable to that of the *Escherichia coli hsd* genes. Mol. Gen. Genet..

[37] Blyn L.B., Braaten B.A., Low D.A. (1990). Regulation of pap pilin phase variation by a mechanism involving differential dam methylation states. EMBO J..

[38] Casadesus J., Maldonado R. (1990). Genomic imprinting in microorganisms. Microbiologia.

[39] Erill I., Puigvert M., Legrand L., Guarischi-Sousa R., Vandecasteele C., Setubal J.C., Genin S., Guidot A., Valls M. (2017). Comparative Analysis of *Ralstonia solanacearum* Methylomes. Front. Plant Sci..

[40] Fang G., Munera D., Friedman D.I., Mandlik A., Chao M.C., Banerjee O., Feng Z., Losic B., Mahajan M.C., Jabado O.J. (2012). Genome-wide mapping of methylated adenine residues in pathogenic *Escherichia coli* using single-molecule real-time sequencing. Nat. Biotechnol..

[41] Oliveira P.H., Ribis J.W., Garrett E.M., Trzilova D., Kim A., Sekulovic O., Mead E.A., Pak T., Zhu S., Deikus G. (2020). Epigenomic characterization of *Clostridioides difficile* finds a conserved DNA methyltransferase that mediates sporulation and pathogenesis. Nat. Microbiol..

[42] Horton J.R., Liebert K., Bekes M., Jeltsch A., Cheng X. (2006). Structure and substrate recognition of the *Escherichia coli* DNA adenine methyltransferase. J. Mol. Biol..

[43] Cohen N.R., Ross C.A., Jain S., Shapiro R.S., Gutierrez A., Belenky P., Li H., Collins J.J. (2016). A role for the bacterial GATC methylome in antibiotic stress survival. Nat. Genet..

[44] Cota I., Bunk B., Sproer C., Overmann J., Konig C., Casadesus J. (2016). OxyR-dependent formation of DNA methylation patterns in OpvABOFF and OpvABON cell lineages of *Salmonella enterica*. Nucleic Acids Res..

[45] Bourgeois J.S., Anderson C.E., Wang L., Modliszewski J.L., Chen W., Schott B.H., Devos N., Ko D.C. (2022). Integration of the *Salmonella* Typhimurium Methylome and Transcriptome Reveals That DNA Methylation and Transcriptional Regulation Are Largely Decoupled under Virulence-Related Conditions. mBio.

[46] Sánchez-Romero M.A., Olivenza D.R., Gutiérrez G., Casadesús J. (2020). Contribution of DNA adenine methylation to gene expression heterogeneity in *Salmonella enterica*. Nucleic Acids Res..

[47] Braaten B.A., Nou X., Kaltenbach L.S., Low D.A. (1994). Methylation patterns in pap regulatory DNA control pyelonephritis-associated pili phase variation in E. coli. Cell.

[48] Braaten B.A., Platko J.V., van der Woude M.W., Simons B.H., de Graaf F.K., Calvo J.M., Low D.A. (1992). Leucine-responsive regulatory protein controls the expression of both the *pap* and *fan* pili operons in *Escherichia coli*. Proc. Natl. Acad. Sci. USA.

[49] Brunet Y.R., Bernard C.S., Gavioli M., Lloubes R., Cascales E. (2011). An epigenetic switch involving overlapping fur and DNA methylation optimizes expression of a type VI secretion gene cluster. PLoS Genet..

[50] Henderson I.R., Owen P. (1999). The major phase-variable outer membrane protein of *Escherichia coli* structurally resembles the immunoglobulin A1 protease class of exported protein and is regulated by a novel mechanism involving Dam and OxyR. J. Bacteriol..

[51] Broadbent S.E., Davies M.R., van der Woude M.W. (2010). Phase variation controls expression of *Salmonella* lipopolysaccharide modification genes by a DNA methylation-dependent mechanism. Mol. Microbiol..

[52] Cowles K.N., Cowles C.E., Richards G.R., Martens E.C., Goodrich-Blair H. (2007). The global regulator Lrp contributes to mutualism, pathogenesis and phenotypic variation in the bacterium *Xenorhabdus nematophila*. Cell Microbiol..

[53] Lanois A., Jubelin G., Givaudan A. (2008). FliZ, a flagellar regulator, is at the crossroads between motility, haemolysin expression and virulence in the insect pathogenic bacterium *Xenorhabdus*. Mol. Microbiol..

[54] Kim J.S., Li J., Barnes I.H., Baltzegar D.A., Pajaniappan M., Cullen T.W., Trent M.S., Burns C.M., Thompson S.A. (2008). Role of the *Campylobacter jejuni* Cj1461 DNA methyltransferase in regulating virulence characteristics. J. Bacteriol..

[55] Shell S.S., Prestwich E.G., Baek S.H., Shah R.R., Sassetti C.M., Dedon P.C., Fortune S.M. (2013). DNA methylation impacts gene expression and ensures hypoxic survival of *Mycobacterium tuberculosis*. PLoS Pathog..

[56] Heithoff D.M., Enioutina E.Y., Daynes R.A., Sinsheimer R.L., Low D.A., Mahan M.J. (2001). *Salmonella* DNA adenine methylase mutants confer cross-protective immunity. Infect. Immun..

[57] Julio S.M., Heithoff D.M., Sinsheimer R.L., Low D.A., Mahan M.J. (2002). DNA adenine methylase overproduction in *Yersinia pseudotuberculosis* alters YopE expression and secretion and host immune responses to infection. Infect. Immun..

[58] Givaudan A., Lanois A. (2000). FlhDC, the flagellar master operon of *Xenorhabdus nematophilus*: Requirement for motility, lipolysis, extracellular hemolysis, and full virulence in insects. J. Bacteriol..

[59] Givaudan A., Lanois A. (2016). Flagellar Regulation and Virulence in the Entomopathogenic Bacteria-*Xenorhabdus nematophila* and *Photorhabdus luminescens*. Curr. Top. Microbiol. Immunol..

[60] Lanois A., Ogier J.C., Gouzy J., Laroui C., Rouy Z., Givaudan A., Gaudriault S. (2013). Draft Genome Sequence and Annotation of the Entomopathogenic Bacterium *Xenorhabdus nematophila* Strain F1. Genome Announc.

[61] Paulick A., Koerdt A., Lassak J., Huntley S., Wilms I., Narberhaus F., Thormann K.M. (2009). Two different stator systems drive a single polar flagellum in *Shewanella oneidensis* MR-1. Mol. Microbiol..

[62] Lobner-Olesen A., von Freiesleben U. (1996). Chromosomal replication incompatibility in Dam methyltransferase deficient *Escherichia coli* cells. EMBO J..

[63] Kovach M.E., Elzer P.H., Hill D.S., Robertson G.T., Farris M.A., Roop R.M., Peterson K.M. (1995). Four new derivatives of the broad-host-range cloning vector pBBR1MCS, carrying different antibiotic-resistance cassettes. Gene.

[64] Gotz S., Garcia-Gomez J.M., Terol J., Williams T.D., Nagaraj S.H., Nueda M.J., Robles M., Talon M., Dopazo J., Conesa A. (2008). High-throughput functional annotation and data mining with the Blast2GO suite. Nucleic Acids Res..

[65] Ondov B.D., Treangen T.J., Melsted P., Mallonee A.B., Bergman N.H., Koren S., Phillippy A.M. (2016). Mash: Fast genome and metagenome distance estimation using MinHash. Genome Biol..

[66] Brillard J., Lereclus D. (2007). Characterization of a small PlcR-regulated gene co-expressed with cereolysin O. BMC Microbiol..

[67] Payelleville A., Blackburn D., Lanois A., Pages S., Cambon M.C., Ginibre N., Clarke D.J., Givaudan A., Brillard J. (2019). Role of the *Photorhabdus* Dam methyltransferase during interactions with its invertebrate hosts. PLoS ONE.

[68] Boemare N.E., Akhurst R.J. (1988). Biochemical and physiological characterization of colony form variants in *Xenorhabdus* spp. (Enterobacteriaceae). J Gen. Microbiol..

[69] Brillard J., Boyer-Giglio M.H., Boemare N., Givaudan A. (2003). Holin locus characterisation from lysogenic Xenorhabdus nematophila and its involvement in Escherichia coli SheA haemolytic phenotype. FEMS Microbiol. Lett..

[70] Jubelin G., Lanois A., Severac D., Rialle S., Longin C., Gaudriault S., Givaudan A. (2013). FliZ is a global regulatory protein affecting the expression of flagellar and virulence genes in individual *Xenorhabdus nematophila* bacterial cells. PLoS Genet..

[71] Pfaffl M.W., Horgan G.W., Dempfle L. (2002). Relative expression software tool (REST) for group-wise comparison and statistical analysis of relative expression results in real-time PCR. Nucleic Acids Res..

