# Supplementary material for: Diverse Roles for a Conserved DNA-Methyltransferase in the Entomopathogenic Bacterium Xenorhabdus
Source: Int J Mol Sci. 2022 Oct 9;23(19):11981. doi: 10.3390/ijms231911981 (PMC9570324; doi:10.3390/ijms231911981)
Supplement: Supplementary file 1 [file ijms-23-11981-s001.zip › ijms-1956018-supplementary.pdf]

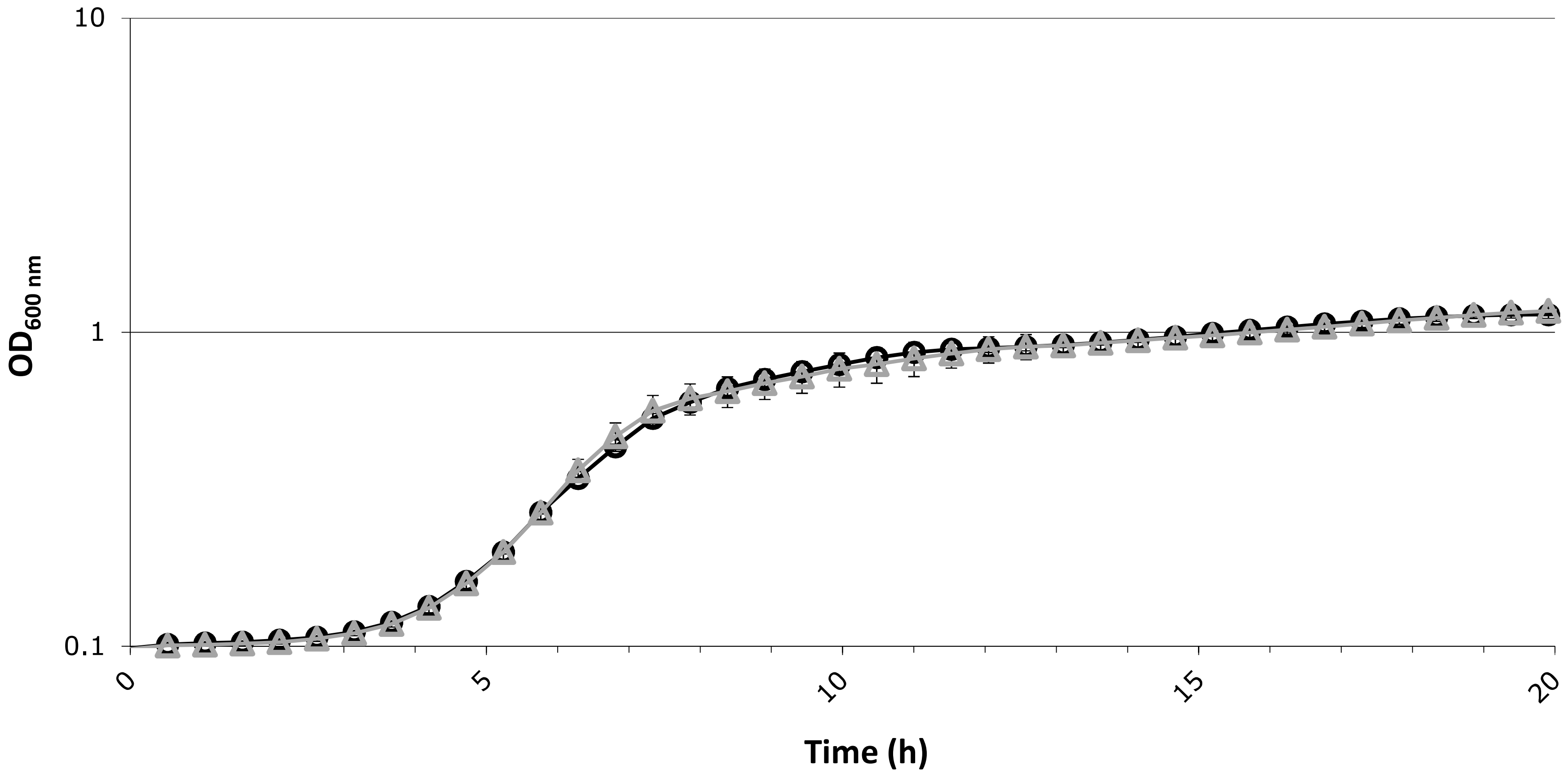

Figure S1. Growth curves of the *X. nematophila dam*-overexpressing strain (grey, triangles) and the control strain (black, circles). Absorbance at 600nm was measured every 30 minutes during growth in LB at 28°C with shaking of the *X. nematophila* Dam-overexpressing strain and the control strain harboring an empty plasmid. Mean values +/- SDs of 3 independent biological replicates for each strain are shown.

Supp data for the Article:

Diverse roles for a conserved DNA-methyltransferase in the entomopathogenic bacterium *Xenorhabdus*

Nadège Ginibre[1=], Ludovic Legrand[2=], Victoria Bientz[1], Jean-Claude Ogier[1], Anne Lanois[1], Sylvie Pages[1], Julien Brillard[1]*

Table S1: Primers used in this study.

<table>
<tr><th>Oligonucleotides</th><th>use</th><th>Sequence (5'- 3')[a]</th></tr>
<tr><td>Cp-dam0322-F</td><td>Cloning of dam gene</td><td>TGCT<u>GAATTC</u>ACAATCTGCGGCTCTGAAAT</td></tr>
<tr><td>Cp-dam0322-R</td><td>Cloning of dam gene</td><td>TGGT<u>GGATCC</u>TGCATTGTTTATCCCCGTTA</td></tr>
<tr><td>qRT-mreB-F</td><td>qRT-PCR on mreB gene</td><td>AATTCTGGTGGGCACTGTTC</td></tr>
<tr><td>qRT-mreB-R</td><td>qRT-PCR on mreB gene</td><td>GGATCGGCTTATCCAACAGA</td></tr>
<tr><td>qRT-recA-F</td><td>qRT-PCR on recA gene</td><td>ATTAATACTCTGGGAGAGTTGATCG</td></tr>
<tr><td>qRT-recA-R</td><td>qRT-PCR on recA gene</td><td>AGTTTCTTATTCAACTCAGCAGCAG</td></tr>
<tr><td>qRT-hsdM-F</td><td>qRT-PCR on XNC3v3_0283 MTase gene</td><td>CGCAAATTAGCTGATGAAAATAGAT</td></tr>
<tr><td>qRT-hsdM-R</td><td>qRT-PCR on XNC3v3_0283 MTase gene</td><td>AGATATTCATAGGCATCACCCATTA</td></tr>
<tr><td>qRT-dam-F</td><td>qRT-PCR on XNC3v3_0322 MTase gene</td><td>CTGATCAATCTGTACAACACCGTAA</td></tr>
<tr><td>qRT-dam-R</td><td>qRT-PCR on XNC3v3_0322 MTase gene</td><td>ATAGCAGTGTCGGTTAAGGTAAAGA</td></tr>
<tr><td>qRT-MTase-1961-F</td><td>qRT-PCR on XNC3v3_1961 MTase gene</td><td>ATCCAAAGCGTATTACAGAAGAAGA</td></tr>
<tr><td>qRT-MTase-1961-R</td><td>qRT-PCR on XNC3v3_1961 MTase gene</td><td>AATACCAATAGCTTCATAGCCTTCC</td></tr>
<tr><td>qRT-MTase-2953-F</td><td>qRT-PCR on XNC3v3_2953 MTase gene</td><td>TGTCAGATGAAAGAGCACCAGT</td></tr>
<tr><td>qRT-MTase-2953-R</td><td>qRT-PCR on XNC3v3_2953 MTase gene</td><td>CGTTAAATCGTTCACGTACCAG</td></tr>
<tr><td>qRT-MTase-3497-F</td><td>qRT-PCR on XNC3v3_3497 MTase gene</td><td>TTAACGTGCTGAACCACATTATCT</td></tr>
<tr><td>qRT-MTase-3497-R</td><td>qRT-PCR on XNC3v3_3497 MTase gene</td><td>TTAGATCTTGGCATTGCTTGTAATA</td></tr>
<tr><td>qRT-MTase-3873-F</td><td>qRT-PCR on XNC3v3_3873 MTase gene</td><td>TGGAAAACAATTCTGGAATTAGAAA</td></tr>
<tr><td>qRT-MTase-3873-R</td><td>qRT-PCR on XNC3v3_3873 MTase gene</td><td>ATTCAGAAGTAAATACGCAGTGTCC</td></tr>
<tr><td>qRT-flhD-F</td><td>qRT-PCR on flhD gene</td><td>CGTTTAGGTATTAGTGAATCGATGG</td></tr>
<tr><td>qRT-flhD-R</td><td>qRT-PCR on flhD gene</td><td>AGATGAGTAGACAGCAAAATACCTG</td></tr>
<tr><td>qRT-fliA-F</td><td>qRT-PCR on fliA gene</td><td>GTAGAACTCGATGACTTGCTTCAG</td></tr>
<tr><td>qRT-fliA-R</td><td>qRT-PCR on fliA gene</td><td>AACTCTTGCTCAAGTTTACGGATG</td></tr>
<tr><td>qRT-fliZ-F</td><td>qRT-PCR on fliZ gene</td><td>GACAAAACCTGGAGTGAATTACAAG</td></tr>
<tr><td>qRT-fliZ-R</td><td>qRT-PCR on fliZ gene</td><td>GACGTCTTAAACGAACCACATACTC</td></tr>
<tr><td>qRT-fliC-F</td><td>qRT-PCR on fliC gene</td><td>GGTATCTCCATTGCTCAGACTACC</td></tr>
<tr><td>qRT-fliC-R</td><td>qRT-PCR on fliC gene</td><td>TGTAGAGATACGGTCAATTTCTTCC</td></tr>
<tr><td>qRT-xaxA-F</td><td>qRT-PCR on xaxA gene</td><td>GAGACAGCAGGAAAATACTCAGAAG</td></tr>
<tr><td>qRT-xaxA-R</td><td>qRT-PCR on xaxA gene</td><td>TGATGGTGGTAGAGAGGTTATTGTC</td></tr>
<tr><td>qRT-xhlA-F</td><td>qRT-PCR on xhlA gene</td><td>GCTGTTGGCAGATAATTCGCC</td></tr>
<tr><td>qRT-xhlA-R</td><td>qRT-PCR on xhlA gene</td><td>CGGATGTGAGTCAGGCTGACA</td></tr>
<tr><td>MSRE-2082-F</td><td>MSRE-PCR on region upstream from XNC3v3_2082</td><td>CAATAGAAGATATTATACCTAATCAACC</td></tr>
<tr><td>MSRE-2082-R</td><td>MSRE-PCR on region upstream from XNC3v3_2082</td><td>GAAAGAAGTTCATTAGTCAATGACAG</td></tr>
</table>

[a] Restriction enzyme sites are underlined
